# A Rare Clinical Pattern: Acquired Dermal Melanocytosis of Nose

**DOI:** 10.1111/jocd.70577

**Published:** 2025-11-28

**Authors:** Batuhan Mustafa Demir, Ferdi Öztürk, Hayriye Sarıcaoğlu

**Affiliations:** ^1^ Department of Dermatology and Venereology Bursa Uludag University School of Medicine Bursa Turkey

**Keywords:** dermal melanocytosis, nasal pigmentation, pigmentation disorders, Q‐switched laser

To the Editor,

Acquired dermal melanocytosis (ADM) is a rare pigmentation disorder that is histopathologically characterized by dermal melanocytes and often occurs in adulthood [1]. Recently, a 25‐year‐old Indonesian woman presented to our outpatient clinic with increased pigmentation around the nose, which started about 5 years ago without any complaint except cosmetic concerns. The patient had no other known diseases and was not taking any regular medications. Dermatologic examination revealed asymmetric irregular pigmented macules with irregular borders on bilateral alar wings, mild pigmentation on the upper lip, and no pigmentation on the oral mucosa or conjunctiva (Figure 1). Dermoscopic examination revealed irregularly distributed light grayish‐brown globules with pseudoreticular character (Figure 2). Skin punch biopsy was recommended to confirm the diagnosis, but the patient did not accept it due to cosmetic concerns; thus, a thin shave biopsy was taken to rule out malignant melanoma. A shave biopsy of the right nasal wing ruled out malignant melanoma with HMB‐45 immunohistochemistry marker being negative. The patient did not have any history of previous application of anything, trauma or lesion in the relevant area in the nasal region. The patient was evaluated as having ADM of the nose based on these clinical and histopathologic features.

**FIGURE 1:**
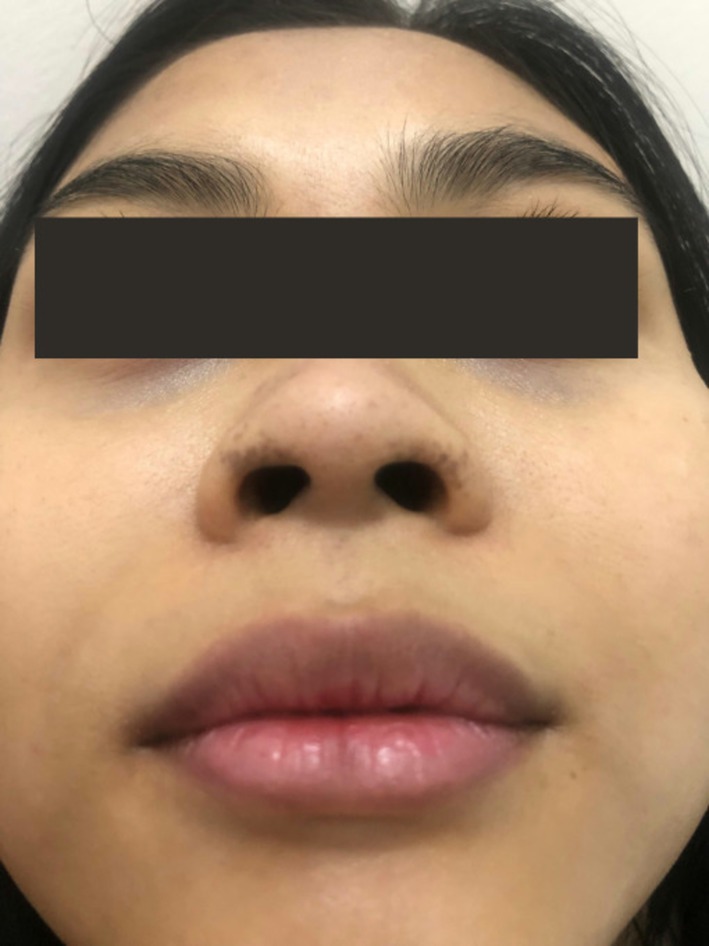
Asymmetric irregular pigmentation on bilateral alar wings, mild pigmentation on the upper lip.

**FIGURE 2:**
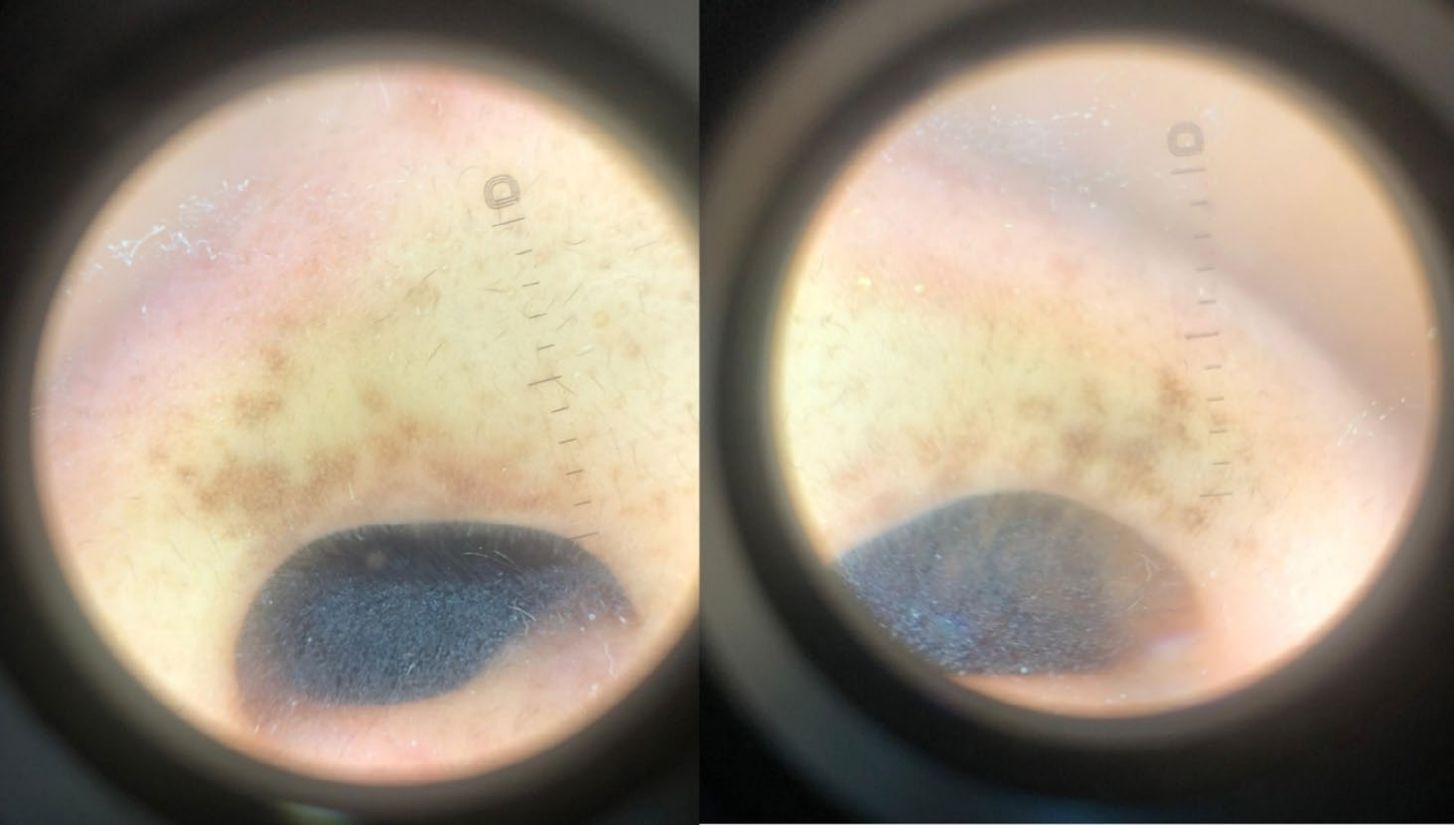
Pseudoreticular distribution of gray‐brown globules on dermoscopy.

Hyperpigmentation disorders can be classified as epidermal, dermal, or mixed epidermal‐dermal hyperpigmentation depending on the location of pigment accumulation. The most common causes of epidermal pigmentation include ephelis, lentigines, café‐au‐lait spots, and pigmentary demarcation lines, which cause a brown appearance. Causes of dermal pigmentation include lichen planus pigmentosus, erythema dyschromicum perstans, pigmented contact dermatitis (Riehl melanosis), and dermal melanocytosis. Dermal hyperpigmentation clinically presents as a gray‐blue color. Postinflammatory hyperpigmentation (PIH) and melasma can be considered as causes of mixed epidermal‐dermal hyperpigmentation. It should also be kept in mind that some topical and systemic medications or heavy metals may also cause pigmentation [2].

Dermal melanocytosis (DM) is a condition characterized histopathologically by dermal melanocytes. Some of it may be present at birth, or some of it develops later in life. It can occur congenitally or be acquired. Congenital dermal melanocytosis (Mongolian spots), Ito nevus, Ota nevus, Hori nevus, blue nevus are among the most common DMs [3, 4]. ADM is a rare and acquired variant of DM. Although it may rarely show extra‐facial involvement, it frequently involves the face. Facial ADM most commonly presents as Hori nevus or acquired bilateral nevus of Ota‐like macules (ABNOM), characterized by bilaterally symmetrical blue‐brown to gray macules of varying pigmentation, often on the cheeks [1, 5]. ADM in the nasal region has been reported in few cases to date. Although it is reported to be more common in Asian women in the literature, this disease may be overlooked or misdiagnosed with other diseases that cause similar clinics, thus causing it to be interpreted as rare in patients other than Asian women [1]. In the differential diagnosis, acquired dermal macular hyperpigmentation, lichen planus pigmentosus, erythema dyschromicum perstans, idiopathic eruptive macular pigmentation, Riehl's melanosis, other dermal melanoses, PIH, melasma, and drug‐induced hyperpigmentation should be considered. In addition to these diagnoses—especially if there is involvement of only one site—melanoma must be ruled out. In case of any doubt, a biopsy should be performed for exclusion [2, 5].

In the treatment of pigmentation disorders, numerous topical, systemic, and procedural treatment methods can be selected based on the etiology of the pigmentation disorder. Since pigmentation in the DM originates from a deeper proportion of the skin compared with the epidermal pigmentation causes, this should be taken into consideration when initiating a treatment. There is no consensus on the treatment to date. Although treatment modalities such as cryotherapy and dermabrasion have been tried, laser treatments are currently prominent in the treatment of DM. Various laser treatments have been tried and found successful in the literature. CO_2_ lasers and Q‐switched (QS) lasers including ruby, alexandrite, and Nd:YAG have been used with successful results in DM treatment [6, 7, 8, 9]. Combination therapies may also be considered for pigmentation disorders. In one study, QS ruby laser and topical treatments were combined in ADM treatment. Researchers applied a combination of 0.1% tretinoin, 5% hydroquinone, and 7% lactic acid to the relevant area 6–8 weeks prior to the QS ruby laser treatment, and a reduction in treatment duration and laser application sessions was observed. This combination may also reduce the risk of PIH development [8]. Studies are ongoing to find the optimal laser treatment to achieve successful treatment outcomes and minimize side effects such as scarring and PIH. There is growing interest in studies comparing different wavelengths to find the ideal laser treatment. In a recent study comparing conventional 1064‐nm QS Nd laser with 730‐nm wavelengths showed that the 730‐nm picosecond laser was found to be more effective in ADM treatment [9].

In our case, the patient was referred to QS laser treatment for ADM of the nose; however, she did not want to undergo the procedure. ADM of the nose, a rare clinical presentation of DM, has very few reports in the literature. We wanted to draw attention to this disease by reporting this rare and special clinical condition.

## Funding

The authors have nothing to report.

## Ethics Statement

Ethical approval was not required for this case report, as it describes the clinical course of a single patient without experimental intervention.

## Consent

Written informed consent was obtained from the patient for publication.

## Conflicts of Interest

The authors declare no conflicts of interest.

## Data Availability

The data that support the findings of this study are available from the corresponding author upon reasonable request.
